# Supplementary material for: Protocol for ICiCLe-ALL-14 (InPOG-ALL-15-01): a prospective, risk stratified, randomised, multicentre, open label, controlled therapeutic trial for newly diagnosed childhood acute lymphoblastic leukaemia in India
Source: Trials. 2022 Jan 31;23:102. doi: 10.1186/s13063-022-06033-1 (PMC8805436; doi:10.1186/s13063-022-06033-1)
Supplement: Supplementary file 1 — Additional file 1: Supplementary Table 1. List of centres participating in the ICiCLe-ALL-14 trial. Supplementary Table 2. The Indian Collaborative Childhood Leukaemia (ICiCLe) study group protocol for risk-stratified treatment of patients aged 1-18 years with first diagnosis of acute lymphoblastic leukaemia (ALL). Supplementary Table 3. SPIRIT item 3. Supplementary Figure 1. Schema of randomisation in the ICiCLe-ALL-14 protocol. [file 13063_2022_6033_MOESM1_ESM.pdf]

Supplementary Data

1. Supplementary Table 1: List of centres participating in the ICiCLe-ALL-14 trial
2. Supplementary Table 2: The Indian Collaborative Childhood Leukaemia (ICiCLe) study group protocol for risk-stratified treatment of patients aged 1-18 years with first diagnosis of acute lymphoblastic leukaemia (ALL)
3. Supplementary Document_ Spirit Item 3
4. Supplementary Document_Toxicity list
5. Supplementary Figure 1: Schema of randomisation in the ICiCLe-ALL-14 protocol
6. Supplementary Document_ WHO Trial Registration Data Set
7. Informed Consent Form
8. Assent Form
9. Patient Information Sheet for BCP-ALL
10. Patient Information Sheet for T-ALL

Supplementary Table 1: List of centres participating in the ICiCLe-ALL-14 trial

Trial Centre	Site	Name	Location
1	1	Department of Pediatric Oncology, Tata Medical Center	Kolkata, India
	2	Department of Medical Oncology, All India Institute of Medical Sciences	New Delhi, India
2	3	Department of Pediatrics, All India Institute of Medical Sciences	New Delhi, India
3	4	Department of Medical Oncology, Tata Memorial Hospital	Mumbai, India
4	5	Department of Pediatrics, Postgraduate Institute of Medical Education and Research	Chandigarh, India
5	6	Department of Medical Oncology, Cancer Institute (WIA)	Chennai, India

Supplementary Table 2: The Indian Collaborative Childhood Leukaemia (ICiCLe) study group protocol for risk-stratified treatment of patients aged 1-18 years with first diagnosis of acute lymphoblastic leukaemia (ALL)

Risk Group	Steroid Prophase	Induction	Consolidation	Interim Maintenance	Delayed Intensification	Maintenance
**Standard**	Days 1-7	Week 2 - 5	Week 6 - 8	Week 9 - 17	Week 18 - 24	Week 25 - 120
B-cell precursor ALL<br>And all of the following<br>NCI Standard Risk<br>Non-bulky disease<br>No high risk genetics<br>No extramedullary sites of disease<br>Good prednisolone response<br>MRD <0·01% at end of induction	Prednisolone 60 mg/m^2 oral	Prednisolone 60 mg/m^2 oral, Day 8-28, & taper<br>Vincristine 1·5 mg/m^2 IV, Day 8, 15, 22, 29<br>*E. coli* L-asparaginase IM, 10,000 IU/m^2 Day 18,21,24, 27<br>IT Methotrexate Day 8, 15, 25	6-mercaptopurine oral 60 mg/m^2, Days 1-21<br>IT Methotrexate Day 8, 15	6-mercaptopurine oral 60 mg/m^2, Days 1-49<br>IT Methotrexate Days 15, 43<br>Methotrexate 20 mg/m^2 oral, Day 1, 8, 22, 29 Day 36, 50, 57<br>Vincristine 1·5 mg/m^2 IV, Day 1, 29<br>Dexamethasone oral 6 mg/m^2, Days 1-5 & Days 29-33	Dexamethasone oral 10 mg/m^2, Days 1-5 & Days 15-19<br>Vincristine 1·5 mg/m^2 IV, Day 1, 8, 15<br>Doxorubicin 25 mg/m^2 IV, Day 1, 8, 15<br>*E. coli* L-asparaginase IM, 10,000 IU/m^2 Day 4, 7, 10, 13<br>IT Methotrexate Days 1, 15<br>Cyclophosphamide IV 1 g/m^2, Day 29<br>Cytarabine 75mg/m^2 IV Days 30-33; 37-40<br>6-mercaptopurine oral 60mg/m^2, Days 29-42	In each 12 week cycle<br>6-mercaptopurine oral 60 mg/m^2, Days 1-84<br>Methotrexate 20 mg/m^2 oral, once a week<br>IT Methotrexate once in 12 weeks (oral methotrexate omitted on week of IT)
**Intermediate**	Days 1-7	Week 2 - 5	Week 6 - 10	Week 11 - 18	Week 19 - 25	Week 26 - 121
B-cell precursor ALL<br>And any of the following<br>NCI High Risk<br>Bulky disease<br>Testicular disease<br>And<br>No high risk genetics	Prednisolone 60mg/m^2 oral	Prednisolone 60 mg/m^2 oral, Day 8-28, & taper (Age ≥ 10 years, pulse Prednisolone Days 8-14 & Days 22-28)<br>Vincristine 1·5 mg/m^2 IV, Day 8, 15, 22, 29	6-mercaptopurine oral 60 mg/m^2, Days 1-28<br>Cyclophosphamide IV 1 g/m^2, Day 1, 15<br>Cytarabine 75mg/m^2 IV<br>Days 2-5; 9-12; 16-19<br>Days 23-26	Methotrexate IV 100 mg/m^2 Day 2<br>Dose increased by 50 mg/m^2 in each of the next treatments<br>Day 12, 22, 32, 42<br>Vincristine 1·5 mg/m^2	As in Standard Risk	As in Standard Risk

No CNS disease<br>Good prednisolone response<br>MRD <0·01% at end of induction		Daunorubicin 25 mg/m^2<br>IV, Day 8, 15<br>*E. coli* L-asparaginase<br>IM, 10,000 IU/m^2<br>Day 9, 12, 15, 18, 21<br>Day 24, 27, 30<br>IT Methotrexate<br>Day 8, 15, 25	IT Methotrexate<br>Day 8, 15	IV, Day 2, 12, 22, 32<br>Day 42<br>IT Methotrexate<br>Day 1, 31		
**High**	Days 1-7	Week 2 - 5	Week 6 - 14	Week 15 - 22	Week 23 - 29	Week 30 - 125
B-cell precursor ALL<br>And any of the following<br>High risk genetics<br>CNS disease<br>Poor prednsolone response<br>MRD ≥0·01% at end of induction	Prednisolone 60mg/m^2<br>oral	Prednisolone 60 mg/m^2<br>oral, Day 8-28, & taper<br>Vincristine 1·5 mg/m^2<br>IV, Day 8, 15, 22, 29<br>Daunorubicin 25 mg/m^2<br>IV, Day 8, 15, 22, 29<br>*E. coli* L-asparaginase<br>IM, 10,000 IU/m^2<br>Day 9, 12, 15, 18, 21<br>Day 24, 27, 30<br>IT Methotrexate<br>Day 8, 15, 25 (CNS disease, extra doses)	6-mercaptopurine oral<br>60 mg/m^2, Days 1-14<br>& Days 29-42<br>Cyclophosphamide IV<br>1 g/m^2, Day 1, 29<br>Cytarabine 75mg/m^2 IV<br>Days 2-5; 9-12; 30-33<br>Days 37-40<br>Vincristine 1·5 mg/m^2<br>IV, Day 16, 23, 44, 51<br>*E. coli* L-asparaginase<br>IM, 10,000 IU/m^2<br>Day 15, 18, 21, 24<br>Day 43, 46, 49, 52<br>IT Methotrexate<br>Day 1, 8, 29	Methotrexate IV 3 g/m^2<br>Day 1, 15, 29, 43<br>IT Methotrexate<br>Day 1, 15, 29, 43<br>6-mercaptopurine oral<br>25 mg/m^2, Days 1-49	As in Standard Risk	As in Standard Risk
**T-lymphoblastic**	Days 1-7	Week 2 -5	Week 6 - 14	Week 15 - 22	Week 23 - 29	Week 30 - 125
**leukaemia / lymphoma**	Prednisolone 60mg/m^2<br>Oral	Dexamethasone oral<br>10mg/m^2 Days 8-14<br>& Days 22-28<br>(replaces Prednisolone)	As in High Risk	Methotrexate IV 5 g/m^2<br>Day 1, 15, 29, 43<br>Rest as in High Risk	As in Standard Risk	As in Standard Risk

		Rest as in High Risk				

NCI, National Cancer Institute, High Risk if presentation white blood cell count ≥ 50×10^9/L and/or age ≥ 10 years at diagnosis
High risk cytogenetics, Hypodiploidy (<40 chromosomes); *BCR-ABL1* fusion, *KMT2A* rearrangement, intrachromosomal amplifcation of chromosome 21, *TCF3-HLF* fusion
CNS disease, cerebrospinal fluid pleocytocis (≥ 5 cells/microlitre) with unequivocal blasts on CSF cytospin; clinical features of CNS disease (e.g. cranial nerve palsies)
Bulky disease, enlargement of liver and/or spleen to the umbilicus or beyond; any single lymph node ≥ 5 cm in maximum diameter; mediastinal mass ≥ 1/3rd of intrathoracic diameter on postero-anterior chest radiograph
Good prednisolone response, absolute circulating blast count < 1000 / microlitre after 7 days of prednisolone monotherapy
MRD, minimal residual disease in bone marrow estimated by 8 or 10-colour flow cytometry
IT, intrathecal (intrathecal methotrexate dose based on age; 1-2 years 8 mg; 2-3 years 10 mg; older than 3 years, 12 mg); IV, intravenous; IM, intramuscular
Asparaginase, where feasible, 1 dose of intramuscular PEG-asparaginase 1000 IU/m^2 replaces 4 doses of native *E. coli* L-asparaginase

Supplementary Document_ Spirit Item 3

A collaborative, multicentre, national study for newly diagnosed patients with acute lymphoblastic leukaemia

ICiCLe ALL-14 Protocol

Issue Date: 7 October 2016

Current version: 5.1

Protocol amendment number: 7

Revision chronology:

Version 1.0, 7 October 2016 Original

Version 2.0, 5 May 2017 Amendment No.1

- Primary reasons for amendment: Change of PI in Tata Memorial Centre, Mumbai; Addition Appendix 20- IAP Body Mass Index Reference Charts_2015; Addition of the word Randomisation 2 in the Consent Form

Version 3.0, 5 Dec 2017 Amendment No.2

- Primary reasons for amendment: T cell ALL Standard Risk group removed from protocol; Details of Cytogenetics testing and Minimal Residual Disease estimation added; Information on risk stratification for CNS disease added

Version 3.1, 15 Mar 2018 Amendment No.3

- Primary reasons for amendment: Addition of Appendix 17- Patient information sheet on Assent; Addition of Appendix 18- Assent form; Addition of Appendix 23-Trial Withdrawal, Treatment Abandonment, Lost to Follow Up

Version 4.0, 3 May 2018 Amendment No.4

- Primary reasons for amendment: Inclusion of MPAL as an exclusion criterion; Recommendation for evaluation of minimal residual disease at the end of

consolidation phase; Categorisation of patients with insufficient risk stratification as High Risk and treatment on trial

Version 4.1, 8 August 2018 Amendment No.5

- Change in Chromosome number for Hypodiploidy

Version 5.0, 25 July 2019 Amendment No.6

- Primary reason for amendment: Replacement of the term “Phase IV” with “Phase III/IV” in type of study

Version 5.1, 7 January 2020 Amendment No.7

- Primary reason for amendment: Modification of study statistical plan

Supplementary Document_Toxicity list

List of CTCAE (version 4.03) Grade 3 – 5 toxicities captured in the trial

Allergy
Avascular necrosis
Bleeding
Constipation/ Ileus
Diarrhoea
Encephalopathy
Hyperglycemia
Hypertension
Infusion-site Extravasation
Oral mucositis
Peripheral neuropathy
Pancreatitis
Psychosis
Seizures
Sepsis
Serum albumin
Serum bilirubin
Serum Creatinine
SIADH
Thromboembolic event
Vomiting

Supplementary Figure 1: Schema of randomisation in the ICiCLe-ALL-14 protocol

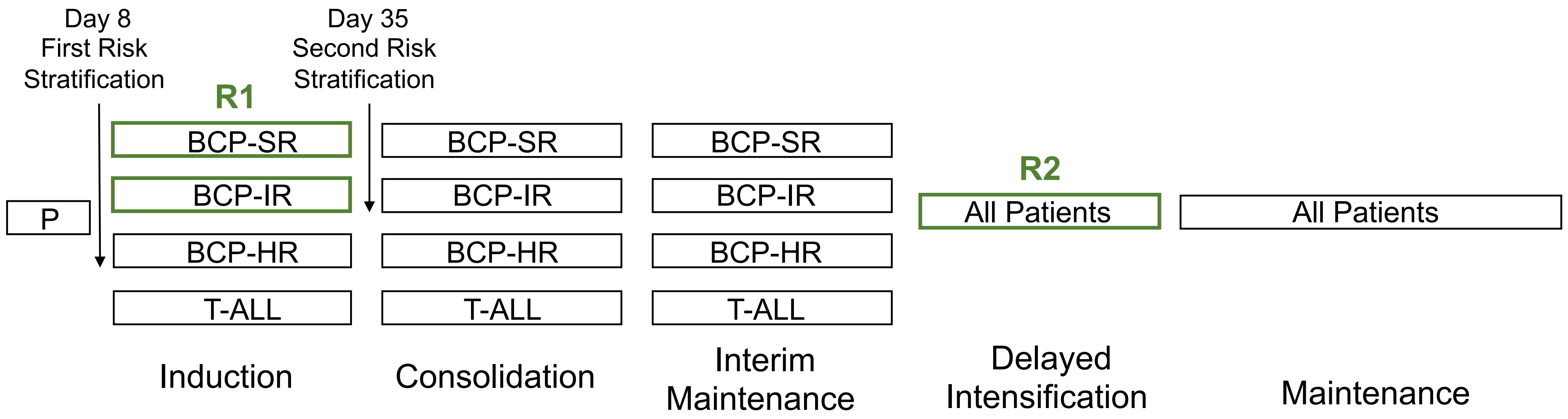

The schema shows the treatment schema of the ICiCLe-ALL-14 protocol. All patients receive 7 days of prednisolone. On day 8, BCP-ALL patients are risk stratified. Patients with a prednisolone poor response and/or high-risk cytogenetics are treated as high risk (HR). Those with a prednisolone good response and non-high-risk cytogenetics are stratified as standard risk (SR) if they are aged <10 years and have a presenting white cell (WC) count <50 x 10^9/L and as intermediate risk (IR) if they are either aged ≥10 years or have a presenting WC ≥50 x 10^9/L. Only SR and IR patients <10 years of age are eligible for the first randomisation of 3 or 5 weeks of steroid therapy. At the end of induction, SR and IR patients who have a MRD ≥10^{-4} (or not evaluable), move to HR stratification. T-ALL and BCP-ALL patients in all risk groups who reach delayed intensification, excluding deaths, withdrawals, refusals and relapse, are eligible for the second randomisation between mitoxantrone and daunorubicin. Consent for randomisations are taken separately before the start of each randomisation.

Supplementary Document_ WHO Trial Registration Data Set

ICiCLe-ALL-14 Trial

1. **Primary Registry**
   Clinical Trials Registry India
   Trial Identifying Number

   CTRI/2015/12/006434

2. **Date of Registration in Primary Registry**
   Registered on 11th December 2015

3. **Secondary Identifying Numbers**
   NIL

4. **Source(s) of Monetary or Material Support**

   Funding: National Cancer Grid (**2016/001)**
   Indian Council of Medical Research (**79/159/2015/NCD-II)**
   DBT-Wellcome India Alliance **(IA/M/12/1/500261)**

   Institutional Support: Tata Medical Center, Kolkata, India
   Tata Memorial Centre, Mumbai, India
   All India Institute of Medical Sciences, New Delhi, India
   Post Graduate Institute of Medical Education & Research, Chandigarh, India
   Cancer Institute Adyar, Chennai, India

5. **Primary Sponsor**
   Tata Medical Center, Kolkata, India

6. **Secondary Sponsor(s)**

   Tata Memorial Centre, Mumbai, India

   All India Institute of Medical Sciences, New Delhi, India
   Postgraduate Institute of Medical Education & Research, Chandigarh, India
   Cancer Institute Adyar, Chennai, India

7. Contact for Public Queries

Dr. Nandana Das

Manager, Clinical Trials Unit

Tata Translational Cancer Research Centre

Tata Medical Center

14 (E-W) Major Arterial Road

New Town, Kolkata 700160

Tel: +91 33 6605 7089

Email: nandana.das@ttcrc.tmckolkata.org

8. Contact for Scientific Queries

Principal Investigator: Professor Vaskar Saha

Director, Tata Tata Translational Cancer Research Center

Tata Medical Center

14 (E-W) Major Arterial Road

New Town, Kolkata 700160

Tel: +91 33 6605 7089

Email: vaskar.saha@ttcrc.tmckolkata.org

9. Public Title

A collaborative, multicentre, national study for newly diagnosed patients with acute lymphoblastic leukaemia

10. Scientific Title

Randomised open label phase IV study for patients with newly diagnosed Acute Lymphoblastic Leukaemia (Indian Childhood Collaborative Leukaemia Group Study ALL 2014)

11. Countries of Recruitment

India

12. Health Condition(s) or Problem(s) Studied

Children with Acute Lymphoblastic Leukaemia

13. Intervention(s)

The details of interventions and comparator agents for randomisation 1 and randomisation 2 are given below in a tabular form.

Type	Name	Details
Randomisation 1	Intervention	3 weeks of pulsed prednisolone during Induction for Standard Risk and Intermediate Risk B-Cell Precursor ALL
	Comparator Agent	5 weeks of continuous prednisolone during Induction for Standard Risk and Intermediate Risk B-Cell Precursor ALL
Randomisation 2	Intervention	One dose of Mitoxantrone during Delayed Intensification in all patients with newly diagnosed ALL
	Comparator Agent	Three doses of Doxorubicin during Delayed Intensification in all patients with newly diagnosed ALL

14. Key Inclusion and Exclusion Criteria

<u>Inclusion Criteria</u>

Age: 1 year to 18 years

Gender: Both

Details: Previously untreated newly-diagnosed ALL including T lymphoblastic lymphoma

<u>Inclusion Criteria</u>

Details: Previously treated patients, patients with Down syndrome and patients with mature B-ALL

15. Study Type

Type of study: Interventional

Study design: Randomized, Parallel Group, Open label, Active Controlled Trial

Phase: III/IV

Sequence generation method: Stratified block randomization

Method of concealment: Centralized

Blinding/Masking: Open Label

16. Date of First Enrollment

25th October 2016

17. Sample Size

Total Sample Size (target): 3056
Current enrolment number: 2301

18. Recruitment Status

Open to Recruitment

19. Primary Outcome(s)

Outcome 1:

Name: Event-free and Overall survival in all patients and within risk groups

Method of analysis: Kaplan-Meier method

The timepoint(s): At end of study and at 3 years from close of study

Outcome 2:

Name: Treatment toxicity in patients treated with 3 weeks of pulsed corticosteroid vs 5 weeks of continuous steroid during Induction in patients with Standard and Intermediate Risk B-Cell Precursor ALL (First randomisation)

Method of analysis: Fisher exact test (for incidence rates between the randomised arms), Poisson regression (for association with potential risk variables such as gender, presentation leucocyte count, disease bulk, cytogenetics, risk group, treatment centre)

The timepoint(s): At end of study

Outcome 3:

Name: Event-free and overall survival in patients treated with 1 dose of Mitoxantrone vs 3 doses of Doxorubicin during Delayed Intensification in all patients (Second Randomisation)

Method of analysis: Kaplan-Meier method

The timepoint(s): At end of study and at 3 years from close of study

20. Key Secondary Outcomes

Outcome 1:

Name: Comparison of complete remission, minimal residual disease and survival (event-free and overall) outcomes between 2 arms in first randomisation

Method of analysis: Fisher exact test, Kaplan-Meier method

The timepoint(s): At end of study and at 3 years from close of study

Outcome 2:

Name: Comparison of treatment toxicity between two arms in second randomisation

Method of analysis: Fisher exact test (for incidence rates between the randomised arms)

The timepoint(s): At end of study

21. Ethics Review

Status: Approved

Date of approval: 7th October 2016 (host centre)

Name and contact details of Ethics committee:

Tata Medical Center-Institutional Review Board (TMC-IRB)
14 (E-W) Major Arterial Road
New Town, Kolkata 700160
Tel: +91 33 6605 7579
Email: irb@tmckolkata.com

22. Completion date

Ongoing study

23. Summary Results

Not applicable (recruitment ongoing)

24. IPD sharing statement

Plan to share IPD: Yes

Plan description: Study data with appropriate de-identification of study participants will be made available and could be accessed from the institutional server at the coordinating trial centre following publication of the results of the study and on reasonable request to the corresponding author.

Tata Medical Center
14 Major Arterial Road (E-W)
New Town, Rajarhat, Kolkata-700 156

Consent Form, ICiCLe ALL-14/INPOG-ALL-15-01 Protocol

Subject Initials: _________

Subject's Name: ______________________________

Date of Birth/ Age: ___/____/_________

MR Number: ___________

Start Date: ___/___/_____

I understand that my child has Acute Lymphoblastic Leukemia and further tests and treatments are needed to determine risk. The treatment will consist of periods of hospitalizations and out-patient visits and will involve the use of chemotherapy and possible radiotherapy. The treatment is intensive and toxic but also associated with a high cure rate. I understand that success is not guaranteed and disease may recur or my child may die of infections associated with the treatment.

The objectives of **ICiCLe ALL-14 study** have been explained to me. I also understand that this is the **'standard of care'** for children with Acute Lymphoblastic Leukemia at this centre.

1. I confirm that I have read and understood the information sheet dated ……………………… for the above study ICiCLe ALL-14 and have had the opportunity to ask questions.

Y	N

2. I give consent for my child to participate in the ICiCLe ALL -14 study

Y	N

3. I have been explained the reasons for randomisation in ICiCLe ALL-14 and I understand that this does not affect the care of my child in any way. I give my consent for my child to participate in the randomisation.

Y	N

Randomisation 1

Y	N

Randomisation 2

I understand that during the process of my child's treatment, data will be collected on my child's progress. This data will be used and published but all my family's details will be anonymised. This data will benefit future patients but probably not my child. If any information acquired will benefit my child or my family, my physician will discuss it with me directly.

4. I give consent for data to be acquired and kept for analysis regarding my child's response to treatment.

Y	N

I understand that at variable time points, blood, bone marrow and other tissue samples will be collected from my child both for diagnosis and monitoring therapy. I understand that Tata Medical Centre, Kolkata will store any extra material obtained from these samples at these times to be used for future ethically approved studies. All materials will be anonymised and the research authorised by appropriate independent review board.

5. I give consent for additional tissue material to be collected and banked from my child.

Y	N

Signing consent does not take the right of withdrawal away. You may withdraw any or one consent obtained at any time. You may choose not to give a reason for your withdrawal from the study. Withdrawal from the study will not affect the care of your child in any way.

Signature (or Thumb impression) of the Subject/Legally Acceptable Representative- ___________________________

Date: __/__/____

Signatory's Name: ___________________________

Signature of the Investigator: ___________________________

Study Investigator's Name: ___________________________ Date: __/__/____

Signature of the Witness: ___________________________

Name of the Witness: ___________________________ Date: __/__/____

Assent form for Children- InPOG ALL 15-01 ICiCLe ALL 14 study

Dear ……………………………………you will be treated for Acute Lymphoblastic Leukaemia, which we tend to call 'ALL'.

We would like to invite you to take part in a study which hopes to improve the way we treat your disease

Background:

To make the disease go away, we will have to give you different medicines which are called chemotherapy. The treatment will last for 2 years. The first 6 months you will have to visit the hospital more often for blood tests, bone marrow tests and your medicines.

1. Why Have I Been Chosen?

We will be asking all children with ALL if they would be willing to take part in this study. The more people that take part, the more information we can gather that will help us in the treatment of children with Acute Lymphoblastic Leukaemia.

2. Do I Have To Take Part?

You do not have to take part in the study. If you and your parents decide to take part, you can still change your minds at any time and your doctor will not mind at all.

3. What Will Happen If I Do Take Part?

You will have intensive chemotherapy for 6 months. Most of this time, we will have will have you come to the hospital and receive your medicines in the Day care. We will admit you in the hospital if you are sick or during some phases of the treatment. You will go to theatre a few times so that we can collect a sample of bone marrow from you. Then you will be treated for 1.5 years with the chemotherapy drugs which you can take by mouth at home. You will have to visit the hospital once in 2 weeks for checking your blood counts and adjust the doses of your medication if needed.

4. Are There Any Other Choices?

Should you or your parents decide not to take part in this study then also your doctor will only treat you on this treatment protocol as this is how we treat all children with Acute Lymphoblastic Leukaemia in our hospital.

5. What Are The Side Effects?

You may feel a bit sick, not feel like eating, have a high temperature and your hair might fall out.

6. What Are The Benefits?

Whatever you and your parents decide, you will only get the very best treatment from your doctors and nurses. We hope that the information we get will make the treatment even better for children like you in the future.

7. Will anyone else know that I am taking part in this study?

The only people who will know that you are taking part in this study will be the team of doctors and nurses looking after you and a group of people who collect information on all the children like you in this study.

8. What will happen to the results of the study?

When the study is finished, the results will be printed in a special sort of newspaper for doctors but your name will not be in it

9. If I have any questions now or later?

If you have any questions, don't be afraid to talk to somebody about them. Doctors and nurses get asked questions all the time, **so** they won't mind.

Thank you for listening to or reading this with a grown up

Patient/Parent Information Sheet

ICICLE ALL-14 clinical study: B-cell precursor acute lymphoblastic leukaemia (BCP-ALL)

An Indian Childhood Collaborative Leukaemia Group Study for Childhood Acute Lymphoblastic Leukaemia

Contents

1. What happens in Acute Lymphoblastic Leukaemia (ALL)?
2. What is the standard treatment for BCP-ALL?
3. Why are we doing this study?
4. Why have my child been chosen for this study?
5. What is new to this study?
6. Are any new medicines being used?
7. What will happen if I take part?
8. What are the benefits of being in this study?
9. What are the possible disadvantages?
10. What happens if I do not wish for randomisation?
11. What happens if I do not wish to take part?
12. What will be done with the study?
13. How is the study monitored?
14. More information

The study

We invite your child to take part in this study. It is important that you understand why this study is being done.

Please take time to read the information carefully. Discuss it with your relatives and friends if you wish. Take your time to decide whether you wish to take part.

Ask your medical team if anything is not clear or if you would like more information.

If you decide to take part, you will be requested to sign a consent form and you will be provided a copy of your signed consent.

The important things you need to know

We wish to find the best treatment with the least side effects for children with BCP-Acute Lymphoblastic Leukaemia.

In the study we are testing two things:

1. A shorter course of steroid therapy than the standard treatment. Steroids are drugs that cause side effects. We hope that by shortening the course of steroids we can reduce the side effects.
2. The benefit of using a drug called Mitoxantrone which has recently been shown by us to be highly effective in treatment of childhood acute lymphoblastic leukaemia.

Regardless of which treatment you choose, treatment will take the same length of time. If you decide not to take part in the study it will not affect the care for you/your child. If you decide initially to take part in the study, you may also later choose to stop taking part.

ICICLE ALL-14 Clinical Study

This study has been reviewed by both Indian and Western experts in childhood acute lymphoblastic leukaemia and approved by ethical committees. All children enrolled in the study will be treated according to a written protocol and will be monitored closely. This ensures that treatment is given correctly and safely. An international independent monitoring committee will closely monitor the safety and outcomes of the study, report to the ethical committees and advise the study coordinators.

1. What happens in Acute Lymphoblastic leukaemia (ALL)?

This study is for children with Acute Lymphoblastic Leukaemia. ALL is the commonest cancer of childhood and affects lymphocytes, a type of white blood cell. In ALL, lymphocytes grow in an uncontrolled way. ALL is of two types, B cell Leukaemia and T cell Leukaemia. The type of ALL will depend on the type of lymphocyte (B cell or T cell) the leukemia cells come from and how mature these leukemia cells are. The uncontrolled growth of abnormal lymphocytes in ALL decreases the production of normal blood cells. An affected child with ALL thus becomes unwell and develops anaemia, easy bleeding and becomes vulnerable to infection. Sometimes the cancer cells in ALL can grow together to form a lump, for example in the chest and cause problems. Untreated, the cells will continue to grow until all bodily functions are halted leading to death.

2. What is the standard treatment for BCP-ALL?

Your child has been diagnosed with B-Cell Precursor Acute Lymphoblastic Leukaemia (BCP-ALL). All patients worldwide are treated with a combination of drugs we call chemotherapy. The treatment has five phases:

- **Induction** - The first 4 weeks is called “induction”.
  Your child will receive 3 to 4 drugs during this time. All children receive weekly Vincristine, Asparaginase and steroids (prednisolone). The latter is usually given over 5 weeks. Some children will additionally receive Daunorubicin. At the end of induction, we expect to no longer see leukaemia cells in the bone marrow and we call this “remission”. We know that we cannot stop treatment at this stage.
- **Consolidation-** This uses additional drugs and lasts 4-6 weeks.
- **Interim Maintenance** - This is a less intensive phase, using fewer drugs and lasts 8 weeks.
- **Delayed intensification** - This is like induction, using similar drugs but a shorter phase. Instead of Daunorubicin we usually treat with Doxorubicin. Treatment is intensified for 7 weeks in this phase.
- **Maintenance** - This uses low dose oral chemotherapy drugs and lasts for a minimum of 2 years.

3. Why are we doing this study?

In the West over 80% of children with BCP ALL can be cured with chemotherapy. Many treatment plans (protocols) are available with similar results. These protocols all use a combination of 10 different chemotherapy drugs and treatment lasts 2-3 years. This intensive treatment has many side effects, including serious infections. As a result many western countries are now evaluating decreasing the intensity of treatment while maintaining high rates of cure.

In most western countries, treatment of childhood ALL is standardised, so a child receives the same standard of care at every centre. This is not so in India and some families travel long distances to centres they perceive offer better treatment.

ICiCLe ALL-14 has two aims:

- Standardise care across the major treatment centres in India.
- Deliver curative treatment that minimises the side effects of therapy in the Indian setting.

4. Why has my child been chosen for this study?

ICiCLe ALL-14 study is for children with ALL. We need to develop better cures for children with ALL in India. Your child has been diagnosed with ALL and we are inviting all children with a diagnosis of ALL in this hospital to be part of this study.

5. What is new in this study?

Although we call this disease ALL, we know that there are different types of ALL. This is based on looking at the chromosome (or genetic structure) changes in leukaemia cells. Unlike normal cell, leukaemia cells have abnormal chromosomes. Some chromosome changes are classified as “low risk”. Patients classed as low risk require minimal treatment for cure. For others classified as “high risk”, we know that more intensive treatment with higher drug doses is required to achieve cure. In the ICiCLe study, we have standardised this approach and treatment will be grouped into “low”, “intermediate” and “high” risk.

Though treatment lasts for over 2 years, we know that the response to treatment in the first 4 weeks can help predict the outcome of treatment. The earlier the disease goes away, the better the outcome of treatment.

In ICiCLe ALL-14 we will look at early treatment response using two techniques at two treatment time points:

- First technique: We look for the presence of leukaemia cells in a blood sample taken 8 days after starting treatment.
- Second technique: We look for the presence of leukaemia cells in the bone marrow using a highly sophisticated technique that we have standardised in ICiCLe ALL-14. This technique allows us to identify 1 leukaemia cell in 10,000 bone marrow cells. This test is called the MRD (minimal residual disease) test. The MRD test is performed at the end of the induction treatment phase (i.e. after 5 weeks of treatment).

We know that those with low level of disease at these two test time points do not need intensive therapy. Those with high levels of disease at these two time points do better with more intensive therapy. In ICiCLe ALL-14, low risk patients receive less intensive treatment and high-risk patients will receive more intensive therapy.

6. Are any new medicines being used?

No new medicines are being used in this study. Firstly, we are investigating whether shortening the duration of steroids will decrease toxicity. The effect of steroid on leukaemia cells is minimal after 5 days. Therefore further use is more likely to increase side-effects. Other groups are investigating this as well and report good results with shorter course of steroids.

Secondly, we are investigating if using Mitoxantrone instead of Doxorubicin will improve treatment results. Both these drugs belong to a family of drugs we call Anthracyclines. Traditionally in ALL we have used the anthracyclines Doxorubicin, Daunorubicin and Idarubicin. Recently we have found that Mitoxantrone is less toxic and is associated with better outcomes when compared to Idarubicin. In ICiCLe ALL-14 we are investigating if it is better than Doxorubicin.

7. What will happen if I take part?

Your child has been diagnosed to have a type of childhood ALL called BCP-ALL. Within 7 days of starting treatment, we will know if your child is in the “low”, “intermediate” or “high” risk group. There are two things that we are testing in ICiCLe ALL-14:

1. During the induction phase, all patients grouped as “low risk” and patients younger than 10 years of age with “intermediate risk” BCP-ALL will be treated with either 5 weeks OR 3 weeks of steroids. Patients classified as high risk will all receive 5 weeks of steroids.
2. During the delayed intensification phase, all low, intermediate and high risk patients will receive either Doxorubicin OR Mitoxantrone. The choice of anthracycline drug will be determined by a process called randomisation.

Short steroid (3 weeks) versus Long steroid (5 weeks) in Induction

Traditionally most western protocols give a steroid called prednisolone for 5 weeks during the induction (first 5 weeks) treatment. This is a very effective drug, but also associated with major side effects, such as serious infection, weight gain, hypertension, disturbances in blood sugar, muscle weakness and bone fractures. There is evidence to suggest we do not need to give steroids continuously for so long, especially in low and intermediate risk patients and centres worldwide are now shortening the period of steroid therapy to decrease side effects.

Mitoxantrone versus Doxorubicin in delayed intensification (between treatment week 18-25)

Mitoxantrone and Doxorubicin belong to the same class of drugs. Doxorubicin has been used traditionally in delayed intensification. We are studying whether Mitoxantrone, which has been commonly used in western protocols, provides a better outcome.

Both the approaches above are standards of care for children with ALL. We are trying to find out which of these would be more suitable for children in India, both to decrease the side effects of treatment and improve cure rates.

Randomisation

The choice of short steroid versus long steroid and the choice of Mitoxantrone versus Doxorubicin will be determined by a process called randomisation. The objective is to divide patients equally into the two treatment groups. This process is necessary to determine which of the two treatments is better for future patients. To avoid any bias, the choice of treatment will be determined by a computer. This choice made by the computer is called **randomisation**. The choice of treatment by randomisation will thus not be influenced by you or your doctor. Randomisation will be discussed with you and your consent will be sought. If you choose not to participate in randomisation, your child will receive conventional treatment with 5 weeks of steroid in induction and with Doxorubicin in delayed intensification.

8. What are the benefits of being in the study?

All clinical studies are carefully designed and monitored. ICiCLe ALL-14 has been designed and reviewed by national and international experts in ALL. A detailed treatment plan (protocol) has been written. Progress and side effects of treatment of every patient will be monitored closely and data shared confidentially by all centres. Combining the information of many patients treated at different centres will allow us to quickly identify where we can do better within the study and treatment of ALL in the future. Due to the close internal and external monitoring, a study is also the safest way to deliver treatment to children with cancer.

9. What are the possible disadvantages?

Irrespective of whether your child takes part in this study, they will all receive chemotherapy. The side effects of chemotherapy include hair loss, infections, mouth ulcers, diarrhoea, changes in appetite and energy, thinning of the bones, and diabetes. Most of these side effects will resolve fairly quickly. In the first randomisation of ICiCLe ALL-14, we are seeking to reduce toxicity and side effects associated with steroid treatment. We have no reason to believe that the steroid randomisation will increase side effects. There is a theoretical possibility that as we are decreasing the intensity of therapy, patients receiving a shorter course of steroids may have a worse outcome. This has been researched thoroughly and we think this is unlikely for a number of reasons:

- The treatment of low risk and intermediate patients is far more intensive in the modern era than in the 1980's; in the 1980's average cure rates reached 80%.
- There is no rationale for the prolonged use of steroids, especially as many different drugs are being used in addition to steroids.

Study outcomes, including early treatment response, serious treatment side-effects, cure rates and differences between the randomised treatments will be examined closely by an independent international data monitoring committee that has the authority to stop the study. Similar studies are also being performed by other groups and we have access to their findings as well. Thus, any adverse outcomes that require halting the study will be detected early.

10. What happens if I do not wish for randomisation?

Your child will be administered conventional treatment. This is 5 weeks of steroids during induction and the drug Doxorubicin during the delayed intensification phase. You may also opt to go ahead with any one of the randomisations or you may opt for none at all, but your child can still can be part of the study.

11. What happens if I do not wish to take part?

Your child will be treated as any other child with ALL, on the non-randomised components of the protocol. We will not collect any data from your child. All other management will remain the same and the treatment of your child will not be compromised in any way.

12. What will be done with the study?

We will obtain your permission to record information on your child's disease and treatment details. To maintain privacy, details of who the information came from will be removed (we call this anonymised data) and it will then be recorded on a computer database. This data will be analysed at regular intervals and the computer is also able to alert the study team if there are any unusual treatment events. At the end of the study, the results

will be published in a medical journal so that doctors worldwide can see the outcome. You may ask your doctor for a copy of any publication. In all publications, your child will remain anonymous.

13. How is the study monitored?

All severe toxicities are reported in a timely manner to the TMC Institutional Review Board. The board reviews these reports regularly and advises if it is safe to continue the treatment. The Institutional Review Board also receives advice from an International Data Monitoring Committee which reviews the progress of the study every 6 months.

As the study provides standard of care, there is no compensation for expected toxicities. The Institutional Review Board may however decide on compensation for events or complications that they feel was a result of other causes.

14. More information

Donating excess material for tissue banking

In addition to taking part of in the study you may be asked to give consent to donate for research purposes, any excess tissue material from diagnostic procedures such as bone marrow tests. You will receive a separate information sheet and consent form for this, but you do not have to agree to this to participate in the study.

What if new information becomes available during the course of the trial?

Sometimes during the study new information becomes available about the medicines being studied. Your doctor will tell you about these development and discuss with you whether it is in your best interests to continue taking part in the study or to move to alternative treatment plans outside the study protocol.

Chief Investigator:
Professor Vaskar Saha

Principal Investigator:
Dr Shekhar Krishnan

Study Local Coordinator:
Dr Nandana Das

Contact:
TATA Medical Center, Kolkata
14 Major Arterial Road (EW)
Rajarhat, New Town
Kolkata 700160
Phone : +91-33-6605-7089
email: icicleall14@tmckolkata.com

Patient/Parent Information Sheet

ICICLE ALL-14 clinical study: T-cell ALL

An Indian Childhood Collaborative Leukaemia Group Study for Childhood Acute Lymphoblastic Leukaemia

Contents

1. What happens in Acute Lymphoblastic Leukaemia (ALL)?
2. What is the standard treatment for T-cell ALL?
3. Why are we doing this study?
4. Why has my child been chosen for this study?
5. What is new to this study?
6. Are any new medicines being used?
7. What will happen if I take part?
8. What are the benefits of being in this study?
9. What are the possible disadvantages?
10. What happens if I do not wish for randomisation?
11. What happens if I do not wish to take part?
12. What will be done with the study?
13. How will the study monitored?
14. More information

The study

We invite your child to take part in this study. It is important that you understand why this study is being done.

Please take time to read the information carefully. Discuss it with your relatives and friends if you wish. Take your time to decide whether you wish to take part.

Ask your medical team if anything is not clear or if you would like more information.

If you decide to take part, you will be requested to sign a consent form and you will be provided a copy of your signed consent.

The important things you need to know

We wish to find the best treatment with the least side effects for children with T-cell Acute Lymphoblastic Leukaemia.

- In the study we are testing the benefit of using a drug called Mitoxantrone which has recently been shown by us to be highly effective in treatment of childhood acute lymphoblastic leukaemia.

Regardless of which treatment you choose, treatment will take the same length of time. If you decide not to take part in the study it will not affect the care for you/your child. If you decide initially to take part in the study, you may also later choose to stop taking part.

ICICLE ALL-14 Clinical Study

This study has been reviewed by both Indian and Western experts in childhood acute lymphoblastic leukaemia and approved by ethical committees. All children enrolled in the study will be treated according to a written protocol and will be monitored closely. This ensures that treatment is given correctly and safely. An international independent monitoring committee will closely monitor the safety and outcomes of the study, report to the ethical committees and advise the study coordinators.

1. What happens in Acute Lymphoblastic leukaemia (ALL)?

This study is for children with Acute Lymphoblastic Leukaemia. ALL is the commonest cancer of childhood and affects lymphocytes, a type of white blood cell. In ALL, lymphocytes grow in an uncontrolled way. ALL is of two types, B cell Leukaemia and T cell Leukaemia. The type of ALL will depend on the type of lymphocyte (B cell or T cell) the leukemia cells come from and how mature these leukemia cells are. The uncontrolled growth of abnormal lymphocytes in ALL decreases the production of normal blood cells. An affected child with ALL thus becomes unwell and develops anaemia, easy bleeding and becomes vulnerable to infection. Sometimes the cancer cells in ALL can grow together to form a lump, for example in the chest and cause problems. Untreated, the cells will continue to grow until all bodily functions are halted leading to death.

2. What is the standard treatment for T-Cell ALL?

Your child has been diagnosed with T-cell Acute Lymphoblastic Leukaemia. All patients worldwide are treated with a combination of anti-cancer drugs we call chemotherapy. The treatment has five phases:

- **Induction** - The first 4 weeks is called “induction”.
  Your child will receive 4 drugs during this time. All children receive weekly Vincristine, Daunorubicin, Asparaginase and Steroids. Steroid treatment may start with Prednisolone and will later switch to Dexamethasone. At the end of induction, we expect to no longer see leukaemia cells in the bone marrow and we call this “remission”. In some cases, we also repeat a chest x-ray at the end of induction to ensure that any chest tumour seen at diagnosis is no longer visible. We know that we cannot stop treatment at this stage.
- **Consolidation-** This uses additional drugs and lasts 9 weeks. New drugs in this treatment phase include Cyclophosphamide, Cytarabine and 6-Mercaptopurine.
- **Interim Maintenance** - This is a less intensive phase and lasts 8 weeks, but requires hospitalisation for administration of high doses of the chemotherapy drug Methotrexate

- **Delayed intensification** - This is like induction, using similar drugs but over a shorter phase. In this phase, Doxorubicin is administered usually instead of Daunorubicin. Treatment is for 7 weeks in this phase.
- **Maintenance** - This uses low dose oral chemotherapy drugs and lasts for a minimum of 2 years.

3. Why are we doing this study?

In the West over 80% of children with BCP ALL can be cured with chemotherapy. Many treatment plans (protocols) are available with similar results. These protocols all use a combination of 10 different chemotherapy drugs and treatment lasts 2-3 years. This intensive treatment has many side effects, including serious infections. As a result many western countries are now evaluating decreasing the intensity of treatment while maintaining high rates of cure.

In most western countries, treatment of childhood ALL is standardised, so a child receives the same standard of care at every centre. This is not so in India and some families travel long distances to centres they perceive offer better treatment.

ICiCLe ALL-14 has two aims:

- Standardise care across the major treatment centres in India.
- Deliver curative treatment that minimises the side effects of therapy in the Indian setting.

4. Why has my child been chosen for this study?

ICiCLe ALL-14 study is for children with ALL. We need to develop better cures for children with ALL in India. Your child has been diagnosed with ALL and we are inviting all children with a diagnosis of ALL in this hospital to be part of this study.

5. What is new to this study?

Patients with T-cell ALL are all treated uniformly across ICiCLe study centres in our country. Though treatment lasts for over 2 years, we know that in T-ALL, the response to treatment at the end of induction (5 weeks) and especially at the end of consolidation (14 weeks) can help predict the probability of cure. The earlier the disease goes away, the higher the probability of cure. In ICiCLe ALL-14, we evaluate this treatment response by using a sophisticated laboratory test called the MRD test (minimal residual disease estimation test). This MRD test has been standardised in ICiCLe ALL-14. The MRD test allows us to identify 1 leukaemia cell in 10,000 bone marrow cells. MRD testing is done both at the end of induction (5 weeks) and at the end of consolidation (14 weeks).

6. Are any new medicines being used?

No new medicines are being used in this study. We are investigating if using Mitoxantrone instead of Doxorubicin will improve treatment results. Both these drugs belong to a family of drugs we call Anthracyclines. Traditionally in ALL we have used the anthracyclines Doxorubicin, Daunorubicin and Idarubicin. Recently we have found that Mitoxantrone is less toxic and is associated with better outcomes when compared to Idarubicin. In ICiCLe ALL-14 we are investigating if it is better than Doxorubicin.

7. What will happen if I take part?

Your child has been diagnosed to have a type of childhood ALL called T-cell ALL. As part of the ICiCLe ALL-14 study treatment for T-ALL, you will be approached for consent to participate in a process called randomisation. This randomisation will be discussed during the delayed intensification treatment phase (treatment weeks 18-25).

As part of randomisation, your child will be administered either Doxorubicin OR Mitoxantrone in the delayed intensification treatment phase. Both drugs belong to the same class of chemotherapy medicines. Doxorubicin has been used traditionally in delayed intensification. We are studying whether Mitoxantrone, which has been used commonly in western protocols, provides a better outcome.

Both drugs, Doxorubicin and Mitoxantrone are standards of care for children with ALL. In ICiCLe ALL-14, we are trying to find out which of the two would be more suitable for children in India, both in terms of cure outcomes and drug side-effects.

Randomisation

The choice of Mitoxantrone versus Doxorubicin will be determined by a process called randomisation. The objective is to divide patients equally into the two treatment groups. This process is necessary to determine which of the two treatments is better for future patients. To avoid any bias, the choice of treatment will be determined by a computer. This choice made by the computer is called **randomisation**. The choice of treatment by randomisation will thus not be influenced by you or your doctor. Randomisation will be discussed with you and your consent will be sought. If you choose not to participate in randomisation, your child will receive conventional treatment with Doxorubicin in delayed intensification.

8. What are the benefits of being in the study?

All clinical studies are carefully designed and monitored. ICiCLe ALL-14 has been designed and

reviewed by national and international experts in ALL. A detailed treatment plan (protocol) has been written. Progress and side effects of treatment of every patient will be monitored closely and data shared confidentially by all centres. Combining the information of many patients treated at different centres will allow us to quickly identify where we can do better within the study and treatment of ALL in the future. Due to the close internal and external monitoring, a study is also the safest way to deliver treatment to children with cancer.

9. What are the possible disadvantages?

Irrespective of whether your child takes part in this study, they will all receive chemotherapy. The side effects of chemotherapy include hair loss, infections, mouth ulcers, diarrhoea, changes in appetite and energy, thinning of the bones, and diabetes. Most of these side effects will resolve fairly quickly.

Study outcomes, including early treatment response, serious treatment side-effects, cure rates and differences between the randomised treatments will be examined closely by an independent international data monitoring committee that has the authority to stop the study. Similar studies are also being performed by other groups and we have access to their findings as well. Thus, any adverse outcomes that require halting the study will be detected early.

10. What happens if I do not wish for randomisation?

Your child will be administered conventional treatment and given the drug Doxorubicin during the delayed intensification phase. You may opt out of randomisation but your child can still can be part of the study.

11. What happens if I do not wish to take part?

Your child will be treated as any other child with ALL, on the non-randomised components of the protocol. We will not collect any data from your child. All other management will remain the same and the treatment of your child will not be compromised in any way.

12. What will be done with the study?

We will obtain your permission to record information on your child's disease and treatment details. To maintain privacy, details of who the information came from will be removed (we call this anonymised data) and it will then be recorded on a computer database. This data will be analysed at regular intervals and the computer is also able to alert the study team if there are any unusual treatment events. At the

end of the study, the results will be published in a medical journal so that doctors worldwide can see the outcome. You may ask your doctor for a copy of any publication. In all publications, your child will remain anonymous.

13. How is the study monitored?

All severe toxicities are reported in a timely manner to the TMC Institutional Review Board. The board reviews these reports regularly and advises if it is safe to continue the treatment. The Institutional Review Board also receives advice from an International Data Monitoring Committee which reviews the progress of the study every 6 months.

As the study provides standard of care, there is no compensation for expected toxicities. The Institutional Review Board may however decide on compensation for events or complications that they feel was a result of other causes.

14. More information

Donating excess material for tissue banking

In addition to taking part of in the study you may be asked to give consent to donate for research purposes, any excess tissue material from diagnostic procedures such as bone marrow tests. You will receive a separate information sheet and consent form for this, but you do not have to agree to this to participate in the study.

What if new information becomes available during the course of the trial?

Sometimes during the study new information becomes available about the medicines being studied. Your doctor will tell you about these development and discuss with you whether it is in your best interests to continue taking part in the study or to move to alternative treatment plans outside the study protocol.

Chief Investigator:
Professor Vaskar Saha

Principal Investigator:
Dr Shekhar Krishnan

Study Local Coordinator:
Dr Nandana Das

Contact:
TATA Medical Center, Kolkata
14 Major Arterial Road (EW)
Rajarhat, New Town
Kolkata 700160
Phone : +91-33-6605-7089
email: icicleall14@tmckolkata.com
